# Supplementary material for: EnzML: multi-label prediction of enzyme classes using InterPro signatures
Source: BMC Bioinformatics. 2012 Apr 25;13:61. doi: 10.1186/1471-2105-13-61 (PMC3483700; doi:10.1186/1471-2105-13-61)
Supplement: Addtional file 5 — The Java code to format the data files, evaluate and predict. The file enzml_java_code.tar.gz contains the Java code used to format database data to ARFF and XML formats, to execute cross and train-test (jackknife) evaluations and to record evaluation results to database. More information is included in the readme.txt file and the Javadoc files. The code can be used with a MySQL database. To use a different database software, other JDBC drivers might be required. [file 1471-2105-13-61-S5.gz › java_code/enzml2011/doc/uk/ac/ed/inf/taweka/test/mulan/attributesfilter/AttributesFilterArffsTest.html]

AttributesFilterArffsTest


---


|  |  |  |
| --- | --- | --- |
| **Overview** \| **Package** \| **Class** \| **Use** \| **Tree** \| **Deprecated** \| **Index** \| **Help** | |  |
| PREV CLASS   **NEXT CLASS** | **FRAMES**    **NO FRAMES**     **All Classes** |  |
| SUMMARY: NESTED \| FIELD \| CONSTR \| METHOD | DETAIL: FIELD \| CONSTR \| METHOD |  |


---


## uk.ac.ed.inf.taweka.test.mulan.attributesfilter Class AttributesFilterArffsTest

```
java.lang.Object
  uk.ac.ed.inf.taweka.test.mulan.attributesfilter.AttributesFilterArffsTest
```

---

``` public class AttributesFilterArffsTest extends java.lang.Object ```

---

| **Field Summary** | |
| --- | --- |
| `static java.lang.String` | `BASE_PROPERTIES_PATH` |
| `static java.lang.String` | `OLDTEST_ARFF_DATA_PROPS` |
| `static java.lang.String` | `TRAIN_ARFF_DATA_PROPS` |


**Constructor Summary**

`AttributesFilterArffsTest()`


| **Method Summary** | |
| --- | --- |
| `int` | `generateArff(java.lang.String dataProps)`             Generate an arff file and return the arff record id |
| `int` | `generateTestArff()`             Generate the old test arff and return the arff record id |
| `int` | `generateTrainArff()`             Generate the train arff and return the arff record id |

**Methods inherited from class java.lang.Object**

`equals, getClass, hashCode, notify, notifyAll, toString, wait, wait, wait`

**Field Detail**

### BASE\_PROPERTIES\_PATH

```
public static java.lang.String BASE_PROPERTIES_PATH
```

---


### TRAIN\_ARFF\_DATA\_PROPS

```
public static java.lang.String TRAIN_ARFF_DATA_PROPS
```

---


### OLDTEST\_ARFF\_DATA\_PROPS

```
public static java.lang.String OLDTEST_ARFF_DATA_PROPS
```


**Constructor Detail**

### AttributesFilterArffsTest

```
public AttributesFilterArffsTest()
```


**Method Detail**

### generateArff

```
public int generateArff(java.lang.String dataProps)
```

:   Generate an arff file and return the arff record id

    :   **Returns:**

---


### generateTestArff

```
public int generateTestArff()
```

:   Generate the old test arff and return the arff record id

    :   **Returns:**

---


### generateTrainArff

```
public int generateTrainArff()
```

:   Generate the train arff and return the arff record id

    :   **Returns:**


---


|  |  |  |
| --- | --- | --- |
| **Overview** \| **Package** \| **Class** \| **Use** \| **Tree** \| **Deprecated** \| **Index** \| **Help** | |  |
| PREV CLASS   **NEXT CLASS** | **FRAMES**    **NO FRAMES**     **All Classes** |  |
| SUMMARY: NESTED \| FIELD \| CONSTR \| METHOD | DETAIL: FIELD \| CONSTR \| METHOD |  |


---
